# An In Vitro Evaluation of the Biological and Osteogenic Properties of Magnesium-Doped Bioactive Glasses for Application in Bone Tissue Engineering

**DOI:** 10.3390/ijms222312703

**Published:** 2021-11-24

**Authors:** Frederike Hohenbild, Marcela Arango Ospina, Sarah I. Schmitz, Arash Moghaddam, Aldo R. Boccaccini, Fabian Westhauser

**Affiliations:** 1 Center of Orthopedics, Traumatology and Spinal Cord Injury, Heidelberg University Hospital, Schlierbacher Landstraße 200a, 69118 Heidelberg, Germany; frederike.hohenbild@med.uni-heidelberg.de (F.H.); sarahisabelle.schmitz@med.uni-heidelberg.de (S.I.S.); 2 Institute of Biomaterials, University of Erlangen-Nuremberg, Cauerstr. 6, 91058 Erlangen, Germany; marcela.arango@fau.de (M.A.O.); aldo.boccaccini@fau.de (A.R.B.); 3 Orthopedic and Trauma Surgery, Frohsinnstraße 12, 63739 Aschaffenburg, Germany; Email@arash.de

**Keywords:** ICIE16 bioactive glass, magnesium, therapeutically active ions, osteogenic differentiation, human mesenchymal stromal cell

## Abstract

Magnesium (Mg^2+^) is known to play a crucial role in mineral and matrix metabolism of bone tissue and is thus increasingly considered in the field of bone tissue engineering. Bioactive glasses (BGs) offer the promising possibility of the incorporation and local delivery of therapeutically active ions as Mg^2+^. In this study, two Mg^2+^-doped derivatives of the ICIE16-BG composition (49.46 SiO_2_, 36.27 CaO, 6.6 Na_2_O, 1.07 P_2_O_5_, 6.6 K_2_O (mol%)), namely 6Mg-BG (49.46 SiO_2_, 30.27 CaO, 6.6 Na_2_O, 1.07 P_2_O_5_, 6.6 K_2_O, 6.0 MgO (mol%) and 3Mg-BG (49.46 SiO_2_, 33.27 CaO, 6.6 Na_2_O, 1.07 P_2_O_5_, 6.6 K_2_O, 3.0 MgO (mol%)) were examined. Their influence on viability, proliferation and osteogenic differentiation of human mesenchymal stromal cells (MSCs) was explored in comparison to the original ICIE16-BG. All BGs showed good biocompatibility. The Mg^2+^-doped BGs had a positive influence on MSC viability alongside with inhibiting effects on MSC proliferation. A strong induction of osteogenic differentiation markers was observed, with the Mg^2+^-doped BGs significantly outperforming the ICIE16-BG regarding the expression of genes encoding for protein members of the osseous extracellular matrix (ECM) at certain observation time points. However, an overall Mg^2+^-induced enhancement of the expression of genes encoding for ECM proteins could not be observed, possibly due to a too moderate Mg^2+^ release. By adaption of the Mg^2+^ release from BGs, an even stronger impact on the expression of genes encoding for ECM proteins might be achieved. Furthermore, other BG-types such as mesoporous BGs might provide a higher local presence of the therapeutically active ions and should therefore be considered for upcoming studies.

## 1. Introduction

The 45S5-bioactive glass (BG) composition (in mol%: 46.1 SiO_2_, 26.9 CaO, 24.4 Na_2_O, 2.6 P_2_O_5_) was introduced by Hench and co-workers in the late 1960s. Since then, a broad variety of BG-compositions has been developed in order to tailor the material properties towards specific needs in bone tissue engineering (BTE) applications [1,2,3,4]. Amongst others, the melt-derived ICIE16-BG composition (in mol%: 49.46 SiO_2_, 36.27 CaO, 6.6 Na_2_O, 1.07 P_2_O_5_, 6.6 K_2_O), developed for the first time in 2004 [5], emerged as a promising candidate. The ICIE16-BG composition offers comparable ion exchange characteristics as the 45S5-BG as its network connectivity (NC) is similar [5,6,7,8]. Furthermore, its larger sintering window results in a lower tendency towards crystallization, which would negatively impact the apatite formation rate [9,10]. Compared to the 45S5-BG composition, ICIE16-BG contains a considerably lower amount of sodium oxide, as the high sodium content of the 45S5-BG induces a strong increase in local pH and is held at least in parts accountable for its dose-dependent cytotoxic effects [11,12,13,14]. A study recently conducted by our group investigated the influence of the ICIE16-BG composition on viability, proliferation and osteogenic differentiation of human mesenchymal stromal cells (MSCs) in a direct co-cultivation setting [13,15]. The ICIE16-BG showed good biocompatibility, was less harmful to cell viability than the 45S5-BG, and stimulated cell proliferation. Regarding the osteogenic potential, the ICIE16-BG significantly outperformed the 45S5-BG at the cellular level, using the alkaline phosphatase (ALP) activity as a correlate of osteogenic differentiation. The influence on the expression of genes encoding for extracellular matrix (ECM) proteins was comparable to the effect of the 45S5-BG [15].

The favorable processing properties of the ICIE16-BG allow the incorporation of therapeutically active ions, which might further enhance its biological capabilities [16]. Therefore magnesium (Mg^2+^)-containing BGs based on the ICIE16-BG composition have been developed, namely 6Mg-BG (composition in mol%: 49.46 SiO_2_, 30.27 CaO, 6.6 Na_2_O, 1.07 P_2_O_5_, 6.6 K_2_O, 6.0 MgO) and 3Mg-BG (composition in mol%: 49.46 SiO_2_, 33.27 CaO, 6.6 Na_2_O, 1.07 P_2_O_5_, 6.6 K_2_O, 3.0 MgO). Since Mg^2+^ ions play an important role in mineral and matrix metabolism [17,18,19,20,21] and have shown to upregulate ECM synthesis [22,23] an improvement of the osteogenic potential might be expected.

In this in vitro study, the biological properties of 3Mg-BG and 6Mg-BG were compared to the ICIE16-BG in order to analyze the biological effect of Mg^2+^ as a part of the ICIE16-BG composition. Therefore, the impact of the BGs on MSC viability, proliferation, osteogenic differentiation and expression of genes encoding for ECM proteins was analyzed in a comparative setting.

## 2. Results

### 2.1. Morphological Characterization and Ion Release Kinetics of BGs

**Figure 1:**
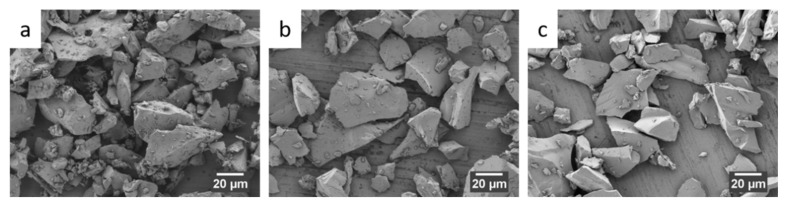
SEM pictures of the bioactive glass (BG) granules. (**a**) ICIE-16 BG, (**b**) 3Mg-BG and (**c**) 6Mg-BG.

Figure 1 shows the typical polyhedral geometry of melt-derived BGs granules used in this study. No differences in terms of morphology are observed for the different BGs.

**Figure 2:**
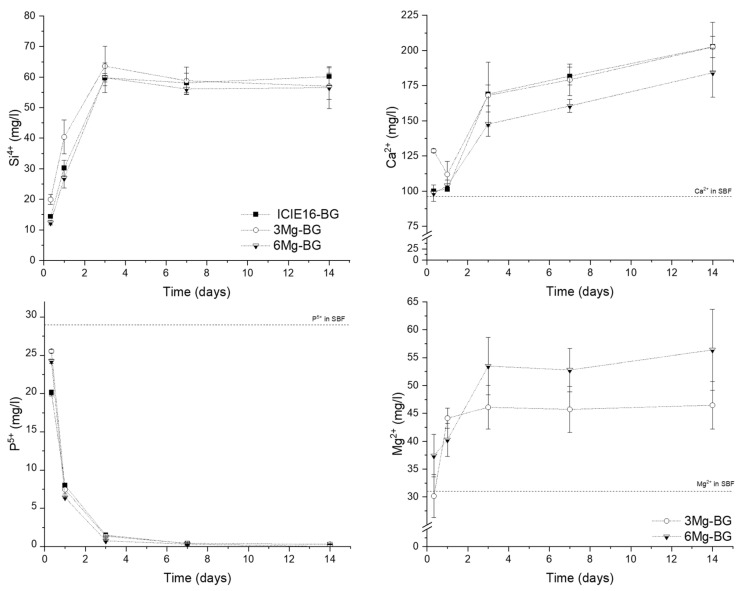
Ion release profiles of bioactive glasses (BGs) as function of incubation time in simulated body fluid (SBF).

The release profiles of released ions from the BGs in simulated body fluid (SBF) are depicted in Figure 2. The amount of Mg^2+^ released from both glasses increased with incubation time. 3Mg-BG exhibited a fast Mg^2+^ liberation during the first day with around 70% of ions released respect to the total amount at 14 days incubation, followed by a slower trend up to 2 weeks. 6Mg-BG supernatant showed a higher Mg^2+^ concentration compared to 3Mg-BG with a rapid release during the first 3 days of incubation with ~30% released during the first day and ~75% after 3 days compared to the total amount at 14 days. The release profiles of Si^4+^, P^5+^ and Ca^2+^ exhibited similar trends between the samples, no significant difference was detected regarding the concentration of ions released from the different BGs, except for the lower release of Ca^2+^ from the 6Mg-BG sample during the first week of incubation.

### 2.2. Mg^2+^-Containing BGs Reduced Cell Proliferation Whilst Showing a Similar Influence on Viability as the ICIE16-BG

**Figure 3:**
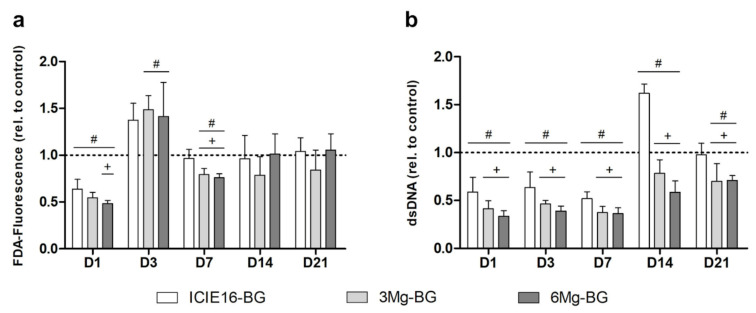
Development of cell viability based on fluorescein diacetate-(FDA-)fluorescence (**a**) and cell proliferation via PicoGreen assay (**b**) from day (D)1 to D21. Results are shown as relative values being normalized to the control group which is depicted by the dotted line. All values are displayed as means with standard deviation. (#) indicates significant difference to the control group and (+) presents significant differences to ICIE16-bioactive glass (BG).

While all BG groups significantly reduced viability compared to the control group on D1, the harmful influence of the BGs was declining with increasing incubation periods, showing no significant differences as of D14. The 6Mg-BG group showed a significantly lower cell viability compared to both control and ICIE16-BG group on D1 and D7, while it was promoting the highest viability on D14 and D21 (Figure 3a).

MSC proliferation was significantly lower in both Mg^2+^-containing BG groups compared to the control during the entire incubation period. The ICIE16-BG group exhibited a significantly lower cell number in comparison to the control until D7, thereafter promoting a strong increase in proliferation and significantly surpassing the control group on D14. The Mg^2+^-supplemented BGs both caused a significantly reduced cell number compared to the ICIE16-BG on all days (Figure 3b).

### 2.3. Earlier Formation of Cell Conglomerates around Granules in the ICIE16-BG Group

**Figure 4:**
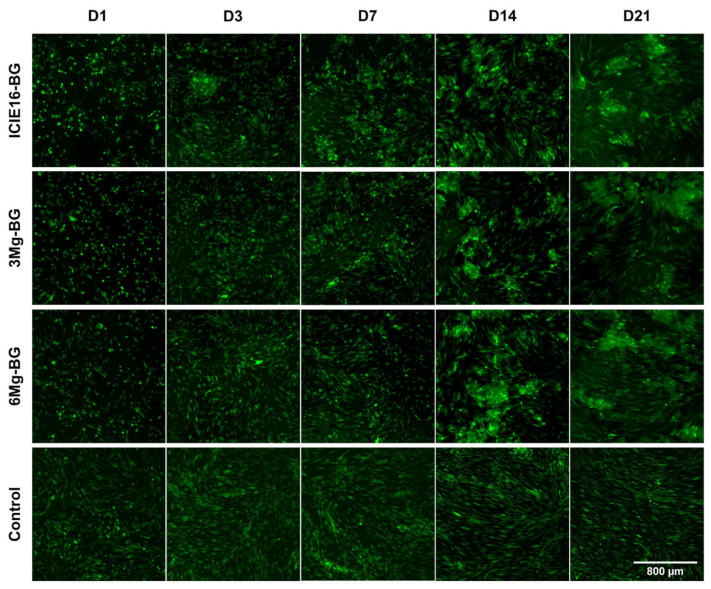
Microscopic presentation of cell viability and growth patterns during 21 days (D) of direct co-culture with bioactive glasses (BGs). Visualization occurred via a live-cell fluorescence staining using fluorescein diacetate (FDA). Magnification: 40-fold; the reference bar of 800 µm applies to all images.

In accordance with the quantitative analysis of viability, microscopic visualization showed a continuously increasing density of MSCs up to D14, thereafter remaining at a stable level. Until D7, the Mg^2+^-containing BGs showed a similar homogenous cell layer as the control group, whereas in the ICIE16-BG group as of D3 cells started adhering to the granules. From D14 on, an increased affinity of the MSCs towards all BGs, leading to the formation of cell conglomerates around the granules, was observed (Figure 4).

### 2.4. Mg^2+^-Containing BGs Partially Enhance Cellular Osteogenic Differentiation and ECM-Related Gene Expression

**Figure 5:**
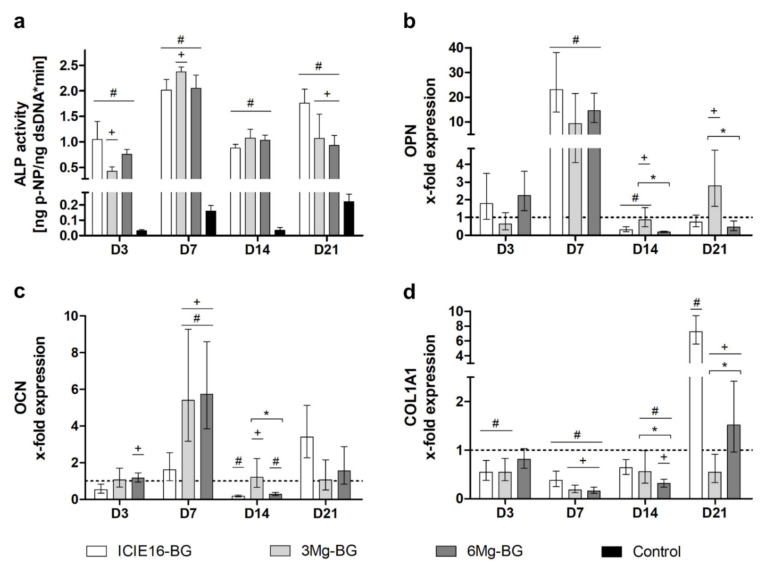
Evaluation of osteogenic differentiation from day (D)1 to D21 represented by alkaline phosphatase (ALP) activity (**a**) and expression of osteopontin (OPN) (**b**), osteocalcin (OCN) (**c**) and type I collagen alpha 1 (COL1A1) (**d**). Relative gene expression was determined by ΔΔCt method: The CT value of each sample was related to glyceraldehyde 3-phosphate dehydrogenase (GAPDH) as endogenous reference gene and subsequently normalized to the control (indicated by a dotted line). Values are shown as means with standard deviation. Significant differences to the control group and the ICIE16-bioactive glass (BG) group are shown by (#) and (+), respectively. (*) designates significant differences between the 3Mg-BG and 6Mg-BG groups encompassed by brackets.

ALP activity was significantly induced by all BGs compared to the control during the entire incubation period. The highest enzymatic activity was observed in the 3Mg-BG group on D7 showing significantly increased values compared to the ICIE16-BG group (Figure 5a). Additionally, the gene expression levels of osteopontin (OPN) and osteocalcin (OCN) were significantly upregulated compared to the control on D7, subsequently declining to expression levels comparable to the initial values. While on D7 the OPN expression was most strongly elevated in the ICIE16-BG group (Figure 5b), the upregulation of OCN was significantly higher in the Mg^2+^-containing BG groups compared to both ICIE16-BG and the control (Figure 5c). In all BG groups the expression of type I collagen alpha 1 (COL1A1) was beneath the control until D14. However, until D21 the expression levels in the ICIE16-BG group rose and exceeded all other groups (Figure 5d).

## 3. Discussion

The known therapeutic effects of Mg^2+^ ions [14,22,24,25,26,27,28,29,30] make them a promising candidate for implementation in different types of biomaterials. BGs present excellent properties to incorporate the therapeutically active ions and allow their controlled local release [31]. While several Mg^2+^-containing BG compositions have been developed, there is still a shortage of studies comprehensively investigating their biological properties.

In the human body Mg^2+^ is the fourth most abundant cation, 50–60% thereof being stored in bone tissue, where it plays an important role in mineral and matrix metabolism [17,18,19,20,21]. A depletion of Mg^2+^ showed to affect all stages of bone metabolism by inhibiting both osteoblast and osteoclast activity, thus leading to osteopenia [19,21,32]. Furthermore, Mg^2+^ is known as a cofactor for more than 300 enzymes and therefore relevant for various processes including synthesis of proteins and nucleic acids, cellular energy metabolism, the regulation of ion channels and intercellular communication [19,26,33,34]. The versatile role of Mg^2+^ in bone remodeling is not yet fully understood, but its involvement in many physiological processes promises good biocompatibility and biological properties [18].

Since the 1870s, Mg^2+^ alloys were explored as implant materials in various fields of application, such as cardiovascular, musculoskeletal and general surgery, and are currently experiencing a rediscovery in biomaterials science [35]. Numerous in vitro studies examined Mg^2+^ containing biomaterials and the effects of their ionic dissolution products observing on the enhancement of cell proliferation and osteogenic differentiation [14,22,24,26,27,28,29,30]. Yoshizawa et al., for instance, examined the influence of MgSO_4_ stimulation on human bone marrow stromal cells detecting both an enhanced production and mineralization of ECM and suggesting Hypoxia-Inducible Factor-2α (HIF-2α) as the Mg^2+^-dependent transcription factor underlying the altered gene expression [22]. Galli and co-workers conducted an in vivo study implanting Mg^2+^-enriched titanium-coated implants in the tibia of rabbits and found a significantly enhanced peri-implant expression of osteogenic marker genes (OCN, runt-related transcription factor 2 (RUNX-2) and insulin-like growth factor I (IGF-1)) as well as the formation of highly mineralized bone tissue around the implants [23]. Considering the material group of BGs, the material characteristics of Mg^2+^-doped BGs have been investigated in numerous studies, showing an improved mechanical stability, lower tendency towards crystallization and high bioactivity [36,37,38]. Nevertheless, the biological effects of the incorporation of Mg^2+^ ions in BG compositions are poorly investigated. Comparable to the above-described impact of Mg^2+^-alloys, Mg^2+^-containing BGs were associated with increased cell proliferation [25,30], enhanced ALP activity [18,24,30] and pronounced expression of osteogenic marker genes [39,40]. However, in some studies the Mg^2+^-doped BGs were only compared to a BG-free control instead of a comparable Mg^2+^-free BG group [18,25] or Mg^2+^ dissolution turned out to be insignificant [39], thus not allowing the specific evaluation of Mg^2+^-related effects.

In the 3Mg-BG and the 6Mg-BG composition investigated in this study, 3 and 6 mol% of CaO, respectively, have been exchanged against MgO. Dissolution in SBF showed that the Mg^2+^ release from both BGs started shortly after contact to the fluid and steadily continued thereafter. As in this setting all BGs were passivated for 24 h before contact to MSCs to reduce their potentially cytotoxic initial bioreactivity, Mg^2+^ is expected to be released to the cell culture medium (CCM) as of the beginning of the culture period.

The stimulating effects of Mg^2+^ ions on cell proliferation were stated in numerous previous studies [22,25,27,29,30], however, a Mg^2+^-related improvement of MSC proliferation could not be observed in this study. Cell numbers in both Mg^2+^-supplemented BG groups were significantly lower compared to the ICIE16-BG group on all days. A known challenge of Mg^2+^ alloys as implant materials is their uncontrolled corrosion in physiological fluids leading to a potentially harmful evolution of hydrogen gas [35,41]. Lozano et al. investigated fluoride surface-modified AZ31 magnesium alloys, assessing a lower proliferation of osteoblasts and a harmful effect on the plasma membrane caused by the direct presence of the alloy [28]. However, Mg alloys and BGs exhibit different degradation behaviors. BG particles can even be used to reinforce Mg alloys leading to a degradable but corrosion resistant composite [42]. As the exchange of CaO against MgO in BGs leads to a lower reactivity a controlled degradation behavior is expected [36] and should thus prevent intolerable rates of Mg^2+^-ion liberation. The above-described release kinetics of the 3Mg-BG and the 6Mg-BG in SBF confirmed the moderate and steady release of Mg^2+^. Therefore, the Mg^2+^ release kinetics are not expected to be fully responsible for the stunted proliferation but a potentiation of BG-induced cytotoxic effects in the enclosed environment of a static in vitro setting might be an explanation.

In contrast to the proliferation behavior, the Mg^2+^-incorporation in the ICIE16-BG had a positive effect on cell viability. On D1 and D7 the fluorometric quantification of viability showed a BG-induced reduction of cell viability in all BG groups, being more pronounced with rising Mg^2+^-supplementation. However, as of D14 higher amounts of Mg^2+^ positively influenced viability and the presence of 6Mg-BG led to a higher cell viability than the unmodified ICIE16-BG. Considering that the cell numbers in the Mg^2+^-supplemented BG groups were lower than in the ICIE16-BG group (as detected in the proliferation assay), it can be concluded, that under the influence of the Mg^2+^ ions the viability of each single cell was increased beyond the effect displayed by the absolute viability measurements. Even though the underlying mechanisms of the participation of Mg^2+^ in bone metabolism are not yet fully understood, Mg^2+^ is known to play an important role in cell metabolism and serves as a cofactor for many enzymes [20,21,33] thereby also influencing cell viability. In this case, cell viability was measured by means of an fluorescein diacetate-(FDA-)based assay depending on the activity of non-specific intracellular esterases [43] that seem to be promoted by the availability of Mg^2+^ ions as reported for different esterases before [44,45,46]. Thus, consistent with the reports about multiple other Mg^2+^-containing BG compositions [14,18,30,38], the Mg^2+^-supplementation sustained the good biocompatibility of the ICIE16-BG showing a superiority of the 6Mg-BG that becomes particularly apparent when regarding viability in relation to cell number.

In the microscopical analysis the Mg^2+^-containing BG groups showed altered growth patterns until D7. While the presence of Mg^2+^-supplemented BGs led to the growth of a consistent cell layer comparable to the control group, MSCs started growing towards the ICIE16-BG granules as of D3. This growth behavior might be caused by the ongoing metamorphosis of the BG surface, as originally described for the 45S5-BG by Greenspan [47]. Upon contact to biological fluids alkali ions and hydrogen exchange rapidly. Initiated by a cascade of reactions a silica-rich surface layer develops and within hours amorphous calcium phosphate precipitates. Crystallization leads to the formation of The HCA layer subsequently formed through crystallization exhibits high similarity to the inorganic mineral phase of bone [47,48]. For the 45S5-BG the presence of an amorphous calcium phosphate layer was observed via X-ray diffraction after reacting in SBF for 8 h, while a fully developed HCA layer was detected after 3 days [49]. Due to the similar NC of the ICIE16-BG the sequence of surface reactions occurs at a comparable rate exhibiting the before mentioned surface characteristics at equivalent time points [8,50]. The exact time sequences of surface transformation in SBF cannot be presumed for a cell culture setting as due to its organic constituents the onset of HCA formation was found to be delayed when biomaterials were immersed in Dulbecco’s modified Eagle’s medium (DMEM) instead of SBF [51,52]. Nevertheless, considering the preceding 24 h passivation period and the microscopically visible affinity of MSCs towards the ICIE16-BG on D3, in this setting the formation of a HCA layer after 4 days of contact to CCM can be assumed. By the incorporation of rising amounts of MgO in a BG composition the formation rate of a MgO-substituted HCA layer is decelerated [36] explaining a later attachment of MSCs to both Mg^2+^-containing BGs. However, from D14 onwards a high affinity of MSCs towards the BG granules was also visible in both Mg^2+^-supplemented groups not indicating a further disadvantage of the delayed cell attachment.

The osteogenic differentiation of MSCs in a conventional in vitro setting can be divided into three major developmental stages, starting with the above described proliferation of cells, followed by the early osteoblastic differentiation and finally passing on to the production and mineralization of an ECM [53,54]. The early cell differentiation is accompanied by a high ALP activity and taking place from day 5 to 14 of incubation [53,54,55]. It should be mentioned that a co-culture of MSCs and BG granules does not fully conform to a conventional cell culture setting, as commonly used BGs create a modified (e.g., alkalized) environment [11,56] and might thus alter the process of cell differentiation. Nevertheless, in this setting on D7 ALP activity was strongly induced by all three BGs with the 3Mg-BG significantly outperforming the reference glass, thus reaching peak activity in the expected period of time. As ALP is expressed by preosteoblasts as well as mature osteoblasts [57], the ALP activity in all BG groups remained continuously elevated thereafter, demonstrating the osteostimulative properties of all three BGs without showing further significant advantages for the Mg^2+^-supplemented BGs.

In the third and final stage, in which the differentiated osteoblasts commit to the formation and mineralization of the ECM, high expression levels of OPN, OCN and COL1A1 are expected [53,57]. OPN expression was significantly induced by all BGs on D7 and the OCN expression levels parallelly peaked on D7 in both Mg^2+^-supplemented BG groups, being significantly higher than the ICIE16-BG group and the control. The COL1A1 expression exceeded its basal levels only on D21, showing a significant induction in the ICIE16-BG group compared to all other groups. Reconsidering the described three stages of osteogenic differentiation a peak in ALP activity is expected before the upregulation of ECM-related genes, while in this study a peak in ALP activity as well as OPN and OCN expression was observed parallelly on D7. However, the determined evaluation time points can only show an extract of the ongoing processes and would thus not capture a peak in gene expression levels that might possibly occur between D7 and D14. Even though all BGs showed promising osteostimulative properties, a clear advantage of the Mg^2+^-doped BGs was only observed regarding ALP activity and OCN expression. Hence, the results of this study do not entirely match the findings in the literature, suggesting the Mg^2+^-related induction of all genes analyzed in this study [23,24,26,29,39]. A possible explanation might be the relatively low release of Mg^2+^ ions from both Mg^2+^-BGs, which would also explain why the differences between both Mg^2+^-supplemented BGs are marginal. The static dissolution profiles in SBF showed that even the 6Mg-BG produced Mg^2+^ concentrations that maximally reached 2.3 mM. Yoshizawa et al. compared the influence of MgSO_4_ at concentrations between 0.8 and 100 mM on cell proliferation, osteogenic differentiation and matrix mineralization and observed the best effects at concentrations of 10 mM [22]. As mentioned above the measurements obtained using SBF as solvent do not allow a direct link to the actual ion concentration in the CCM. Nevertheless, they can give an orientation and might thus suggest that the amount of Mg^2+^ ions released from the 3Mg-BG as well as the 6Mg-BG was not high enough to reveal the full therapeutic potential of the Mg^2+^ ions. As in this study MSCs were brought in direct contact to the BG granules, it is not possible to distinguish physical from chemical effects. To assess the effects of the ionic dissolution products in insolation, an indirect culture setting would be necessary. However, as the physical contact plays a crucial role in the cell-material interaction [14,58], in this study a direct culture setting was chosen to better simulate the more complex in vivo situation and thus explore the BGs’ potential for future in vivo studies. Due to the moderate amount of released Mg^2+^ ions, it must be assumed that at least parts of the observed effects were not exclusively induced by the dissoluted Mg^2+^ ions but also the combination of the BGs’ physical and chemical effects. In order to further benefit from the ion supplementation, the release kinetics of Mg^2+^ ions from the BGs should be reconsidered. An attractive alternative to moderately deliver a sufficient amount of Mg^2+^ ions is the incorporation in mesoporous BGs (MBGs). Owing to their high surface area and pore structure MBGs offer excellent release kinetics for the local delivery of bioactive substances [59,60,61,62]. While therapeutically active ions can be incorporated into the framework of MBGs, the mesoporous structure can simultaneously serve as a loading efficient vector for local drug delivery [59,60]. A recent study conducted by Tabia et al. demonstrated that by Mg^2+^-doping the specific surface area of MBGs can be further enhanced and thereby lead to improved drug release kinetics [63]. Thus, Mg^2+^-doped MBGs might be promising candidates to benefit of potentially synergistic effects of a combined local release of Mg^2+^ ions and other therapeutically active substances (e.g., drugs [64,65,66,67] or growth factors [68,69,70]).

For a deeper understanding of the role of Mg^2+^ as a component of BGs further investigation will be necessary. For instance, this study examined the influence on ECM production only on a gene expression level, leaving the question how the actual production of ECM proteins and the subsequent mineralization were influenced. Further interesting aspects of investigation are the effect of Mg^2+^-doped BGs on cell adhesion [71,72] and anti-inflammatory processes [27], which are both assumed to be influenced by Mg^2+^ but were not yet examined in the context of Mg^2+^-supplemented BGs.

## 4. Materials and Methods

### 4.1. BG Production and Ion Release

The glasses were produced from mixtures of analytical grade reagents, namely, NaCO_3_ (Honeywell Fluk, Steinheim, Germany), K_2_CO_3_ (Alfa Aesar, Erlenbachweg, Germany), CaCO_3_ (Honeywell Fluka), CaHPO_4_·_2_H_2_O (Acros Organics, Geel, Belgium), MgO (Sigma-Aldrich, Steinheim, Germany) and commercial-grade Belgian quartz sand (SiO_2_). The batches were melted in Pt crucibles at 1420 °C for 1.5, followed by casting in graphite molds and annealing at 520 °C for 1 h. Subsequently, the glasses were crushed and ground to powders with a planetary ball mill (Retsch, Haan, Germany) and sintered at 690 °C for 1.5 h. Morphological characterization of the granules was done with scanning electron microscopy at 1.5 kV (SEM, Auriga, Carl-Zeiss, Jena, Germany).

For ion release measurements, 75 mg of glass particles were immersed in 50 mL of SBF as described in previous studies [73] and incubated in an orbital shaker at 37 °C and 90 rpm agitation. After different incubation times, the SBF supernatant was filtered and analyzed with an inductively coupled plasma optical emissions spectrometer (ICP-OES, PerkinElmer Optima 5300 DV, Shelton, CT, USA) to obtain the concentration of ions released from the glasses.

### 4.2. Study Ethics and Cell Origin

MSCs of n = 10 patients undergoing surgery at the proximal femur for medical reasons at the Heidelberg Orthopedic University Hospital were harvested. Donor cells were used to establish a cell pool in order to compensate donor-dependent differences in cell behavior, as described previously [74]. Prior to cell extraction written consent was granted from all patients. The responsible ethics committee of the Medical Faculty of the University of Heidelberg approved the study (S-443/2015).

### 4.3. MSC Isolation, Cultivation and Characterization

MSCs were extracted from freshly collected bone marrow and cultivated as described previously [74,75,76]. Cells were washed in phosphate-buffered saline (PBS; Life Technologies, Darmstadt, Germany) and afterwards cultivated in expansion medium composed of 83% DMEM high glucose supplemented with 12.5% FCS, 1% L-glutamine, 1% non-essential amino acids (NEAA; all Life Technologies), 1% penicillin/streptomycin (Biochrom, Berlin, Germany), 0.1% β-mercaptoethanol (Life Technologies) and 4 ng/mL fibroblast growth factor 2 (Abcam, Cambridge, UK). Medium was first changed after 24 h to wash out non-adherent cells. Subsequently medium was changed twice weekly until a confluency of 80% was achieved. The individual donor cells were pooled in passage 1 following previously published recommendations [74]. Cells were stored in liquid nitrogen until usage in passage 5.

### 4.4. General Experimental Design: Overview

Prior to introduction to the cell culture setting, BGs were sterilized at 160 °C for 30 min in an Heraeus Function Line heating and drying oven (Heraeus instruments, Hanau, Germany) and passivated in DMEM for 24 h following recent recommendations [11,56]. The passivated BG granules were subjected to CCM (89% DMEM high glucose, 10% FCS, 1% penicillin/streptomycin). BG-containing media were placed in 24- and 96-well culture plates (both Nunc, Roskilde, Denmark) and MSCs were seeded aiming for a cell density of 1.8 × 10^4^ cells per cm^2^ and a final BG concentration of 2.5 mg/mL. MSCs cultured in BG-free CCM served as control. Twice weekly, media of all groups were replaced by fresh CCM.

**Figure 6:**
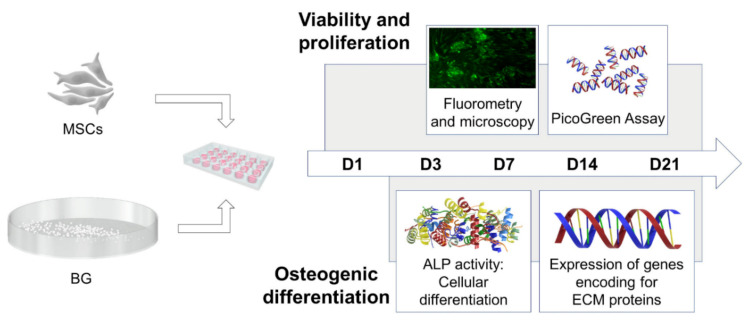
Overview of the experimental design. Mesenchymal stromal cells (MSCs) were co-cultured with bioactive glass (BG) granules for a total incubation period of 21 days (D). On D1, D3, D7, D14 and D21 cell viability was analyzed quantitatively as well as qualitatively and cell proliferation was assessed. In addition, alkaline phosphatase (ALP) activity and expression of genes encoding for extracellular matrix (ECM) proteins were evaluated starting from D3 after incubation.

The influence on cell viability and proliferation was evaluated both qualitatively and quantitatively on day 1 (D1), 3 (D3), 7 (D7), 14 (D14) and 21 (D21). The impact of Mg^2+^ on the differentiation of MSC towards osteoblasts was analyzed by evaluation of ALP activity [75,76] and the influence of Mg^2+^ on the ECM production was quantified by expression analysis of genes encoding for ECM proteins on D3, D7, D14 and D21, as schematically depicted in Figure 6.

### 4.5. Microscopical and Fluorometric Analysis of Cell Viability and Proliferation

To simultaneously allow quantitative and microscopic assessment of cell viability an (Sigma-Aldrich) staining was conducted. FDA freely passes the cell membrane being intracellularly hydrolyzed by nonspecific esterases generating the green-fluorescent fluorescein, whose fluorescence intensity is correlating with the cell viability [43,77,78]. In 96-well culture plates, supernatants were discarded and cells washed in PBS. An amount of 100 µL of 10 µg/mL FDA staining solution were added to each well and incubated for 5 min at 37 °C and 5% CO_2_. To determine cell morphology and growth patterns, the wells were visualized using an Olympus IX-81 inverted fluorescence microscope (Olympus, Hamburg, Germany). For simultaneous quantification of cell viability, stained cells were washed in PBS and afterwards lysed in 150 µL 0.5% Triton X-100 (Sigma-Aldrich) for 5 min. Lastly fluorescence intensity was determined at a wavelength of 535 nm using a Wallac 1420 Victor microplate reader (Perkin Elmer, Waltham, MA, USA).

Proliferation was analyzed using the Quant-iT PicoGreen dsDNA Assay Kit (Life Technologies) as described in the manufacturer’s instructions. The fluorochrome PicoGreen selectively binds double-stranded DNA (dsDNA) and thus permits the quantification of mononuclear cells such as MSCs.

### 4.6. Assessment of ALP Activity as a Correlate of Osteoblastic Development

In order to determine ALP activity, a commonly used colorimetric assay based on para-nitrophenylphosphate (p-NPP) was applied following established protocols [74,75,76]. ALP catalyzes the hydrolyzation of p-NPP to the chromogenic para-nitrophenol (p-NP), allowing the analysis of enzymatic activity of ALP, which is proportional to the extinction of the yellow compound. Cells were lysed in 500 µL 1% Triton X-100 and stored at −80°C until further use. Fifty microliters of each sample were combined with 50 µL ALP buffer (0.1 M glycine (Carl Roth, Karlsruhe, Germany), 1 mM ZnCl2, 1 mM MgCl_2_ (both Merck); pH 10.4), and 100 µL p-NPP substrate (1 mg/mL; Sigma-Aldrich) and incubated for 90 min at 37 °C. The extinction, measured in a PHOMO microplate reader (Autobio Diagnostics, Zhengzhou, China) at a wavelength of 405/492 nm, was used to calculate the ALP activity. Each value was normalized to the corresponding dsDNA content.

### 4.7. qPCR of Genes Encoding for ECM Proteins

For isolation of total RNA the PureLink RNA Mini Kit (Life Technologies) was utilized as described by the manufacturer. To synthesize complementary DNA (cDNA) the High-Capacity RNA-to-cDNA Kit (Life Technologies) was used as specified in the manufacturer’s protocol. Real-time quantitative polymerase chain reaction (RT-qPCR) was performed to examine the expression of relevant members of the osseous ECM, being OCN, OPN and COL1A1. The respective primers are listed in Table 1. For calculation of the relative gene expressions the ΔΔCt method was applied, relating each target gene to glyceraldehyde 3-phosphate dehydrogenase (GAPDH) as an endogenous reference gene and subsequently normalizing the expression to the control group. The RT-qPCR was conducted using SYBR Green Master Mix (Life Technologies).

### 4.8. Statistics

IBM SPSS Statistics (Version 25; IBM, Armonk, NY, USA) was used to test values via one-way ANOVA followed by Bonferroni’s post-hoc test accepting *p*-values of <0.05 as significant. GraphPad Prism (Version 8.1.0; GraphPad Software, La Jolla, CA, USA) was applied to create graphs. N = 5 biological replicates were used for all experiments and measured in technical duplicates. Results are depicted as rounded means with standard deviation.

## 5. Conclusions

In this study, the influence of two Mg^2+^-doped derivatives of the ICIE16-BG on viability, proliferation and osteogenic differentiation of MSCs was compared to the original ICIE16-BG composition in order to determine the biological effects of a local Mg^2+^ release. All examined BGs were biocompatible in direct co-culture to MSCs. While cell viability was positively influenced by the Mg^2+^-incorporation, proliferation was significantly lower in both Mg^2+^-supplemented BGs. Regarding the osteogenic properties, no overall superiority for either of the BGs could be determined. The Mg^2+^-incorporation exhibited a positive influence on ALP activity and OCN expression, whereas the induction of COL1A1 expression was higher without the presence of Mg^2+^. The moderate effects on the activity of genes encoding for relevant ECM proteins is most likely caused by a comparably low Mg^2+^ ion release from the BGs. Other BG-types that might provide a higher local presence of the therapeutically active ions such as mesoporous BGs should be considered for the local delivery of Mg^2+^ in future studies.

## Figures and Tables

**Table 1:** Primers deployed for qPCR: glyceraldehyde 3-phosphate dehydrogenase (GAPDH; reference gene), osteocalcin (OCN), osteopontin (OPN) and type I collagen alpha 1 (COL1A1).

Gene	Forward (5′ ⟶ 3′)	Reverse (3′ ⟶ 5′)
GAPDH	GCC CAA TAC GAC CAA ATC AGA GA	GAA AGC CTG CCG NGT GAC TAA
OCN	ACC GAG ACA CCA TGA GAC CC	GCT TGG ACA CAA AGG CTG CAC
OPN	GCT AAA CCC TGA CCC ATC TC	ATA ACT GTC CTT CCC ACG GC
COL1A1	GTG GCC TGC CTG GTG AG	GCA CCA TCA TTT CCA CGA GC

## Data Availability

All relevant data are available within the paper.
